# Sociodemographic characteristics of patients throughout the recruitment process into a randomized, controlled behavioral trial

**DOI:** 10.1186/s13063-025-09225-7

**Published:** 2025-11-18

**Authors:** Deborah Rohm Young, Melissa Preciado, Viviann Soto, Edith Fauresviun, Jennifer J. Jimenez, Margo A. Sidell, Justin N. Tayag, Freddy Arriola, Silvia R. Paz, Deborah A. Cohen, Anny H. Xiang, May L. Wang, Maureen O.’Keeffe Rosetti, Stephen P. Fortmann

**Affiliations:** 1 Department of Research & Evaluation, Kaiser Permanente Southern California, 100 S. Los Robles, Pasadena, CA 91101 USA; 2 Center for Health Research, Kaiser Permanente Northwest, 3800 N. Interstate Ave, Portland, OR 97227 USA

**Keywords:** Recruitment, Sociodemographics, Randomized trial, Behavioral intervention

## Abstract

**Background:**

Recruiting participants into trials is important to ensure trial success. We report the results of a recruitment process using electronic health records (EHR) to identify potentially eligible patients with telephone follow-up to enroll participants into a randomized, controlled trial of a physical activity intervention.

**Methods:**

Patients aged 18 to 77 years with prediabetes or diabetes not using insulin and reporting low physical activity were potentially eligible. An EHR program code was created to assess eligibility. Patients received an email or mailed letter explaining the trial and information that they will be contacted in the coming weeks. Recruiters made up to 5 contact attempts. Patients were categorized as never reached, contacted by phone, consented, and randomized. Information was collected from the EHR to identify sociodemographic characteristics through the recruitment stages. Pearson’s chi-squared test and Fisher’s exact test were used to test for differences in categorical variables; Wilcoxon rank sum test was used for continuous variables. Analyses were completed using R version 4.3.0.

**Results:**

Recruitment continued from July 2020 through September 2023 with a goal of 482 participants. A total of 11,152 patients received either an email (89.5%) or letter (10.5%) informing them of potential eligibility (pre-diabetes: 66%; female: 57%; Hispanic: 65%; Spanish-language preference: 25%; neighborhoods with a high deprivation index: 24%). Recruiters contacted 4033 patients by phone; those contacted were more likely to have prediabetes than diabetes (77%), to be female (61%), were less likely to be Hispanic (60%), or to have a Spanish-language preference (17%) compared to those not contacted by phone (all *p* < 0.001). Among those contacted by phone, patients who consented (*N* = 721) differed from those who did not: they were more likely to be pre-diabetic (87%), female (75%), and less likely to prefer Spanish language (10%) (all *p* < 0.001). Four hundred fifty-one were randomized; 23% lived in neighborhoods with a high deprivation index, which did not differ from those not randomized.

**Conclusions:**

Following a recruitment process of patients identified in the EHR, people with diabetes, males, Hispanics, and Spanish speakers were less likely to participate in a physical activity behavior trial for patients with diabetes or prediabetes, with no apparent differences in neighborhood deprivation.

**Trial registration:**

Randomized Trial of Exercise Promotion in Primary Care NCT0445168. Registered on June 24, 2020.

**Supplementary Information:**

The online version contains supplementary material available at 10.1186/s13063-025-09225-7.

## Background

Participant recruitment is a key factor for success in randomized clinical trials. A sufficient number of participants are needed to achieve adequate power [1]. For trial results to be generalizable beyond the trial sample, participants should be representative of the underlying population [2]. Recruitment strategies can influence the ultimate generalizability of the trial’s findings.

Active versus passive recruitment methods differ in their strategy as well as their ability to identify individuals most likely to benefit from the treatment. Passive methods, such as distributing flyers or mailers to people within certain ZIP codes, can reach a large number of people with minimal expense. These methods may, however, require extensive screening to determine if eligibility criteria are met [3]. Active methods that include reaching out directly to people who may meet eligibility criteria are more costly but may ultimately result in participants who are more representative of the underlying population of interest.

It is generally not reported how representative participants are to the underlying population of those potentially eligible. Buffenstein and colleagues, in a systematic review and meta-analysis, reported on the representativeness of 2977 clinical trials with respect to reporting gender, race, and ethnicity [4]. While gender is consistently reported, only 53% reported race and 36% reported ethnicity. If demographic information is not reported in clinical trials, neither is it likely that readers are able to learn about the demographic make-up of those who were contacted to be recruited. Without this information, the generalizability of trial participants cannot be correctly discerned. This paper describes the methods used to recruit participants into a behavioral physical activity trial and to identify and compare the sociodemographic characteristics of patients as they moved through the recruitment stages and to describe differences across stages. The trial relied on active methods.

## Methods

The Exercise Promotion in Primary Care (EPPC) trial was a randomized, controlled trial to assess the effects of a 2-year motivational interviewing intervention on moderate-to-vigorous physical activity among adults with prediabetes or type 2 diabetes not being treated with insulin. Potential participants received their primary care at one large medical center in Southern California. After obtaining informed consent and following the completion of baseline assessments, participants were randomized to either the motivational interviewing intervention or healthy lifestyle materials that were distributed via email at 6-week intervals. The trial was approved by the Kaiser Permanente Southern California (KPSC)/Hawaii Institutional Review Board (IRB #10106). Trial recruitment occurred from July 2020 to September 2023, with a target goal of 482 participants. Recruitment, data collection, and intervention activities were remotely completed, as was originally planned but was fortuitous due to the COVID-19 pandemic occurring during this time.

Eligible patients were those who recently had an outpatient visit at the medical center and were between the ages of 18 and 77 years [5, 6]. They must have had a diagnosis of diabetes but were not prescribed insulin or a prediabetes diagnosis or a HbA1c value between 5.7 and 6.4%, fasting glucose between 100 and 125 mg/dL, or oral glucose tolerance test between 140 and 199 mg/dL recorded in their electronic health record (EHR). Additional inclusion criteria included being able to speak and write in English or Spanish, having participated in less than 60 min of moderate physical activity per week as determined by self-report [7], having a body mass index between 18.5 and 40 kg/m^2^, willingness to be randomized, and having access to a telephone. Exclusion criteria were mostly medical exclusions that may have made it unsafe for a patient to increase their physical activity. These included a cardiovascular event within the prior 6 months, cancer diagnosis or treatment within 1 year, psychiatric hospitalization in the prior 2 years, heart failure, history of uncontrolled hypertension, thyroid, or seizures, a blood condition that increased the risk of bleeding, end-stage renal disease, peripheral vascular disease, muscle, joint, or bone problems that limit the ability to be physically active, among other rare conditions. Other exclusions include currently pregnant or breastfeeding or plans to become pregnant in the next 2 years, concurrent participation in other clinical trials or research programs, current participation in any exercise or weight loss program, and plans to discontinue KPSC membership or leave the area in the next 2 years.

An EHR program code was created to identify potentially eligible patients to be invited to participate in EPPC. Key data, such as preferred spoken/written language, weight, diagnosis, and primary care provider, were extracted from the EHR and imported into REDCap (Research Electronic Data Capture) [8, 9] to support and streamline recruitment. The program detected seemingly eligible patients who had an outpatient visit within the previous 7 days. The body mass index range was expanded to 17.4 to 47.7 kg/m^2^ in the program to allow for weight to change during the recruitment process. After about one week, patients were sent one email in either English or Spanish, depending on the patient’s preference. The email briefly described the trial, stated they may be eligible and that they would receive a phone call from a study staff member to further explain the trial and determine interest. An opt-out link was included if the patient did not wish to receive a follow-up phone call. A subset of patients received a letter by mail if an email contact was not available. The list was capped at 75 patients per week so recruiters had a reasonable workload. Any excess from a list was used when the weekly patient list did not meet 75.

Recruiters were bilingual and bicultural in English and Spanish and trained in the trial’s activities. Patients received up to 5 calls over a 2-week period across different days of the week and times of day, leaving no more than 5 voice mails. REDCap was utilized to document key details of each recruitment call, including the start and end times, whether the recruiter successfully spoke with a participant, left a voicemail, or scheduled a callback. Unsuccessful call attempts were also recorded, such as instances where no voicemail could be left, the number was disconnected, or someone other than the intended participant answered. Additional documentation included the participant’s interest in the study, completion of the study questionnaire, and consent to receive text messages. If the patient was not able to be reached after these attempts, they were classified as a passive decliner. When a patient was reached, after describing the study and if a patient was interested in learning more, a series of screening questions were administered to confirm eligibility. Included in the screening questions was a verification of spoken and written language to ensure that study materials were aligned with their language preference. Interested and eligible patients with diabetes were cleared for participation by a primary care provider prior to consent. In all but a small number of instances, the patient was cleared to participate. For patients with prediabetes, they were asked if they had about 10 more minutes to provide informed consent over the phone. Patients were sent an online consent form to review while the recruiter further explained that if they participated in the trial, they would be randomized into either the motivational interviewing arm or usual care, what would be expected of them if they participated in the trial, and obtained informed consent. Patients were informed that within the following 2 weeks, they would receive a packet containing the following items: an introduction letter, a waist-worn accelerometer and belt, instructions on how to wear the accelerometer, an accelerometer log, a hard-copy survey questionnaire, and a return UPS envelope.

After obtaining consent, patients were contacted by measurement staff to describe in detail how to wear the accelerometer over 7 consecutive days and how to complete the short baseline survey either online or by paper. After completion of baseline assessments, depending on patient preference, they were sent a $25 gift card. Patients were subsequently contacted by phone and asked again to verify their willingness to participate in the trial and be randomized to either treatment or usual care. If they confirmed their willingness to participate, they were randomized. Randomization procedures included stratification by race and ethnicity (Hispanic, White, Other), sex, and diabetes status (prediabetes, diabetes). Within strata, randomization was generated in blocks of varying sizes to assure balance across treatment arms over time.

Sociodemographic variables were identified from the KPSC EHR system. Age was calculated from date of birth. Race and ethnicity were categorized as non-Hispanic white (White), non-Hispanic black (Black), Hispanic, Asian and Pacific Islander (Asian), and other, multiple races or ethnicities, and unknown (other/unknown). Sex was classified as male or female. Patients confirmed their preferred spoken language recorded in the EHR. If no preferred language was recorded, it was updated in REDCap.

Using geocoded patient’s home addresses, a neighborhood deprivation index (NDI) was created based on the methods of Messer et al. [10]. To create the index, socioeconomic and demographic characteristics, which includes the constructs of income/poverty, education, employment, housing, and crowding, were compiled at the census tract level from the US Census American Community Survey (ACS). Quintiles were formed based on ACS information from census tracts populated by all KPSC patients.

### Statistical analyses

Analyses were conducted to identify potential differences by age, diagnosis, sex, race and ethnicity, language preference, and neighborhood deprivation, as patients went through the recruitment process. We analyzed those who responded and those who did not at each recruitment stage. Categorical variables are displayed as *N* and percentages, continuous variables as means ± standard deviations (SD). Pearson’s chi-squared test and Fisher’s exact test were used for categorical variables, and the Wilcoxon rank sum test was used for continuous variables to test for differences between groups. Analyses were completed using R version 4.3.0.

## Results

Between July 2020 and July 2023, a total of 11,152 patients received either an email (89.5%) or letter (10.5%) informing them that they may be eligible to participate in EPPC. There were 346 patients who opted out of the study at the email link. About two-thirds (66%) met the criteria for pre-diabetes; the remainder had diabetes not prescribed insulin (Table 1). More females (57%) than males were contacted, and most (65%) were of Hispanic ethnicity. 25% of total contacted patients preferred Spanish as their spoken language based on information recorded in the EHR. Of those who were contacted by phone, 59% had their primary residence in locations that were classified in the two most deprived neighborhoods based on the NDI.

**Table 1:** Demographic information for adults meeting eligibility criteria based on electronic health record information who were contacted by email or mail, subsequently contacted by phone, or not contacted by phone

Characteristic	Total *N* = 11,152^1^	Contacted by phone *n* = 4033^1^	Not contacted by phone *n* = 7119^1^	*P* value^2^
Diabetes status				< 0.001
Pre-diabetes	7274 (66%)	3104 (77%)	4170 (59%)	
Diabetes	3806 (34%)	915 (23%)	2891 (41%)	
Age, years, mean (SD)	54.6 (11.8)	53.1 (12.2)	55.5 (11.5)	< 0.001
Sex				< 0.001
Female	6348 (57%)	2449 (61%)	3899 (55%)	
Male	4804 (43%)	1584 (39%)	3220 (45%)	
Race and ethnicity				< 0.001
Hispanic	7195 (65%)	2429 (60%)	4766 (67%)	
White	1610 (14%)	654 (16%)	956 (13%)	
Black or African American	1365 (12%)	598 (15%)	767 (11%)	
Asian and Pacific Islander	520 (4.7%)	179 (4.4%)	341 (4.8%)	
Other/unknown	462 (4.1%)	173 (4.1%)	289 (4.1%)	
Preferred spoken language				< 0.001
English	8306 (74%)	3364 (83%)	4942 (69%)	
Spanish	2835 (25%)	669 (17%)	2166 (30%)	
Neighborhood deprivation index				< 0.001
1—least deprived	465 (4.2%)	208 (5.2%)	257 (3.7%)	
2	1103 (10%)	429 (11%)	674 (9.6%)	
3	2634 (24%)	1003 (25%)	1631 (23%)	
4	4177 (38%)	1433 (36%)	2744 (39%)	
5—most deprived	2605 (24%)	916 (23%)	1689 (24%)	

^1^*n* (%) unless otherwise noted

^2^Pearson’s chi-squared test Fisher’s exact test for categorical measures or Wilcoxon rank sum test

Displayed in Table 1, study recruiters were able to contact 4033 patients by phone. Compared to those who were not able to be contacted, those contacted by phone were more likely to meet the criteria for prediabetes, be female, and be White or Black, and were less likely to be Hispanic and patients who preferred Spanish as their spoken language (all *p* < 0.001). Those who lived in the least deprived neighborhoods were more likely to be contacted by phone compared with those living in the most deprived neighborhoods (*p* < 0.001).

Patients who consented to participate in EPPC (*N* = 721) differed from those who did not consent (*N* = 3312) (Table 2) by the demographic differences that were similar to the results in Table 1. Of note, those who consented compared with individuals who did not consent were more likely to be classified as pre-diabetic (87% vs 75%), be younger (mean age 51.2 [SD: 11.1] vs 53.5 [SD: 12.2]), be female (75% vs 58%), and state that English was their preferred language (90% vs 82%) (all *p* values < 0.001). There was no statistically significant difference in NDI for those who consented and those who did not.

**Table 2:** Demographic information for adults meeting eligibility criteria based on electronic health record information who were contacted by phone, consented to participate in EPPC, and who did not consent to participate

Characteristic	Contacted by phone *n* = 4033^1^	Consented to participate *N* = 721^1^	Did not consent to participate *n* = 3312^1^	*P* value^2^
Diabetes status				< 0.001
Pre-diabetes	3104 (77%)	628 (87%)	2476 (75%)	
Diabetes	915 (23%)	91 (13%)	824 (25%)	
Age, years, mean (SD)	54.0 (12.2)	51.2 (11.7)	53.5 (12.2)	< 0.001
Sex				< 0.001
Female	2449 (61%)	541 (75%)	1908 (58%)	
Male	1584 (39%)	180 (25%)	1404 (42%)	
Race and ethnicity				< 0.001
Hispanic	2429 (60%)	416 (58%)	2013 (61%)	
White	654 (16%)	116 (16%)	538 (16%)	
Black or African American	598 (15%)	139 (19%)	459 (14%)	
Asian and Pacific Islander	179 (4.4%)	16 (2.2%)	163 (4.9%)	
Other/unknown	173 (4.1%)	34 (4.7%)	139 (4.2%)	
Preferred spoken language				< 0.001
English	3364 (83%)	646 (90%)	2718 (82%)	
Spanish	669 (17%)	75 (10%)	594 (18%)	
Neighborhood deprivation index				0.30
1—least deprived	208 (5.2%)	46 (6.4%)	162 (4.9%)	
2	429 (11%)	83 (12%)	346 (11%)	
3	1003 (25%)	177 (25%)	826 (25%)	
4	1433 (36%)	240 (34%)	1193 (36%)	
5—most deprived	916 (23%)	170 (24%)	746 (23%)	

^1^*n* (%) unless otherwise noted

^2^Pearson’s chi-squared test Fisher’s exact test for categorical measures or Wilcoxon rank sum test

Ultimately, 451 of the patients who consented completed the baseline assessment and were randomized into EPPC (62.6% of consented) (Table 3), achieving 93.6% of the target of 482 participants. Of the 11,152 patients contacted by email or mail, this represents a 4.0% yield, and an 11.2% yield from those who were reached by telephone. Compared to patients who consented but were not randomized, those who were randomized were older (52.6 [SD: 11.3] vs 49.9 [SD: 11.9]). A slightly higher percentage of patients randomized had diabetes than those not randomized, although this was not statistically significant (*p* = 0.07). All other sociodemographic variables did not differ.

**Table 3:** Demographic information for adults meeting eligibility criteria based on electronic health record information who consented to participate in EPPC, were randomized, and were not randomized

Characteristic	Consented to participate *N* = 721^1^	Randomized to participate *N* = 451^1^	Were not randomized *n* = 270^1^	*P* value^2^
Diabetes status				0.07
Pre-diabetes	628 (87%)	386 (86%)	242 (90%)	
Diabetes	91 (13%)	65 (14%)	26 (9.7%)	
Age, years, mean (SD)	51.2 (11.7)	52.6 (11.3)	49.0 (11.9)	< 0.001
Sex				
Female	541 (75%)	346 (77%)	195 (72%)	
Male	180 (25%)	105 (23%)	75 (28%)	
Race and ethnicity				0.30
Hispanic	416 (58%)	251 (56%)	165 (61%)	
White	116 (16%)	79 (18%)	37 (14%)	
Black or African American	139 (19%)	88 (20%)	51 (19%)	
Asian and Pacific Islander	16 (2%)	8 (1.8%)	8 (3.0)	
Other/unknown	34 (5%)	25 (5.5%)	9 (3.3%)	
Preferred spoken language				0.60
English	646 (90%)	406 (90%)	240 (89%)	
Spanish	75 (10%)	45 (10%)	30 (11%)	
Neighborhood deprivation index				0.30
1—least deprived	46 (6%)	33 (7.4%)	13 (4.9%)	
2	83 (12%)	53 (12%)	30 (11%)	
3	177 (25%)	118 (26%)	59 (22%)	
4	240 (34%)	140 (31%)	100 (37%)	
5—most deprived	170 (24%)	104 (23%)	66 (25%)	

^1^*n* (%) unless otherwise noted

^2^Pearson’s chi-squared test Fisher’s exact test for categorical measures or Wilcoxon rank sum test

**Fig. 1:**
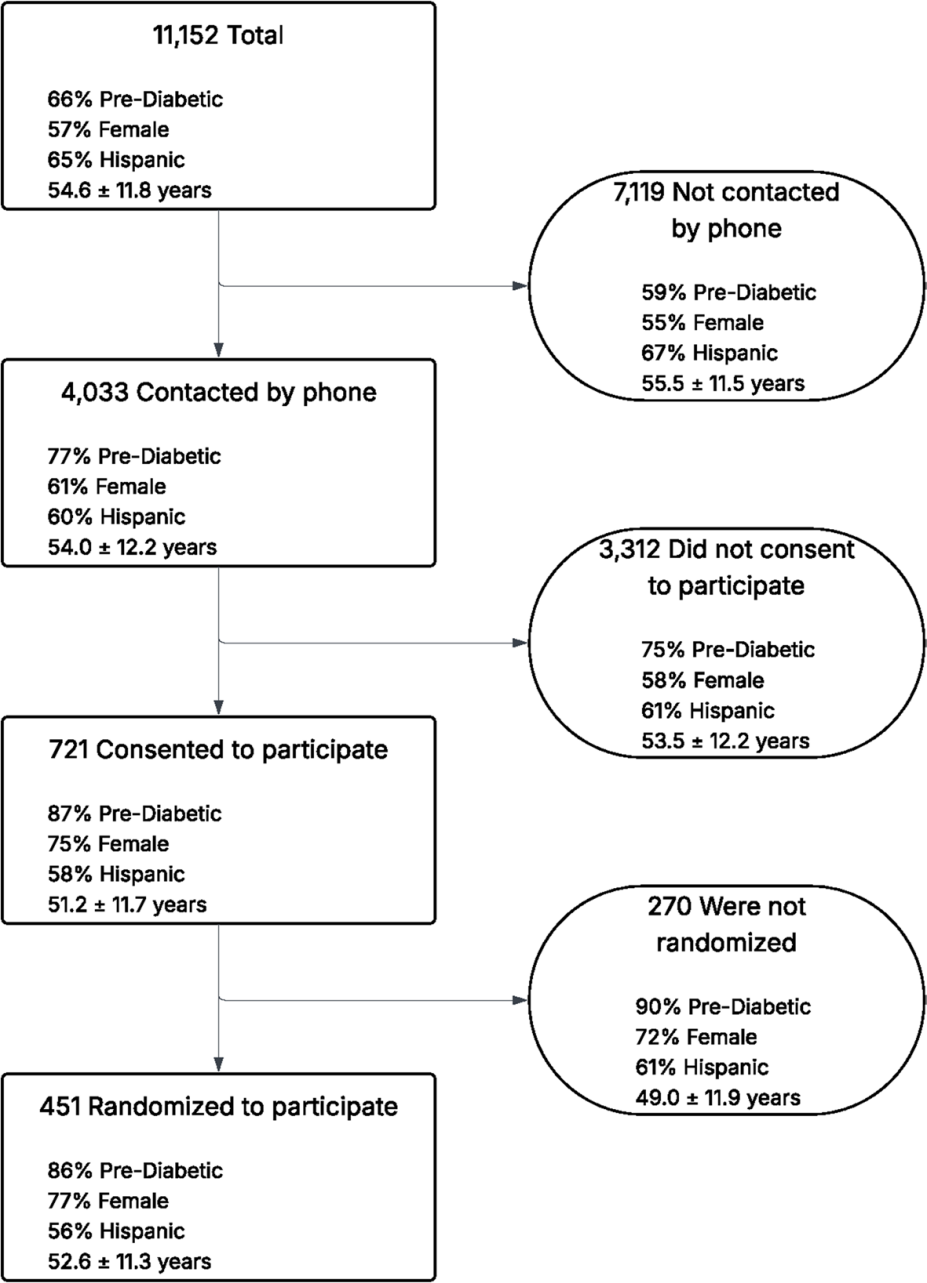
Recruitment flow of sociodemographic information of participants from initial outreach to randomization

**Fig. 2:**
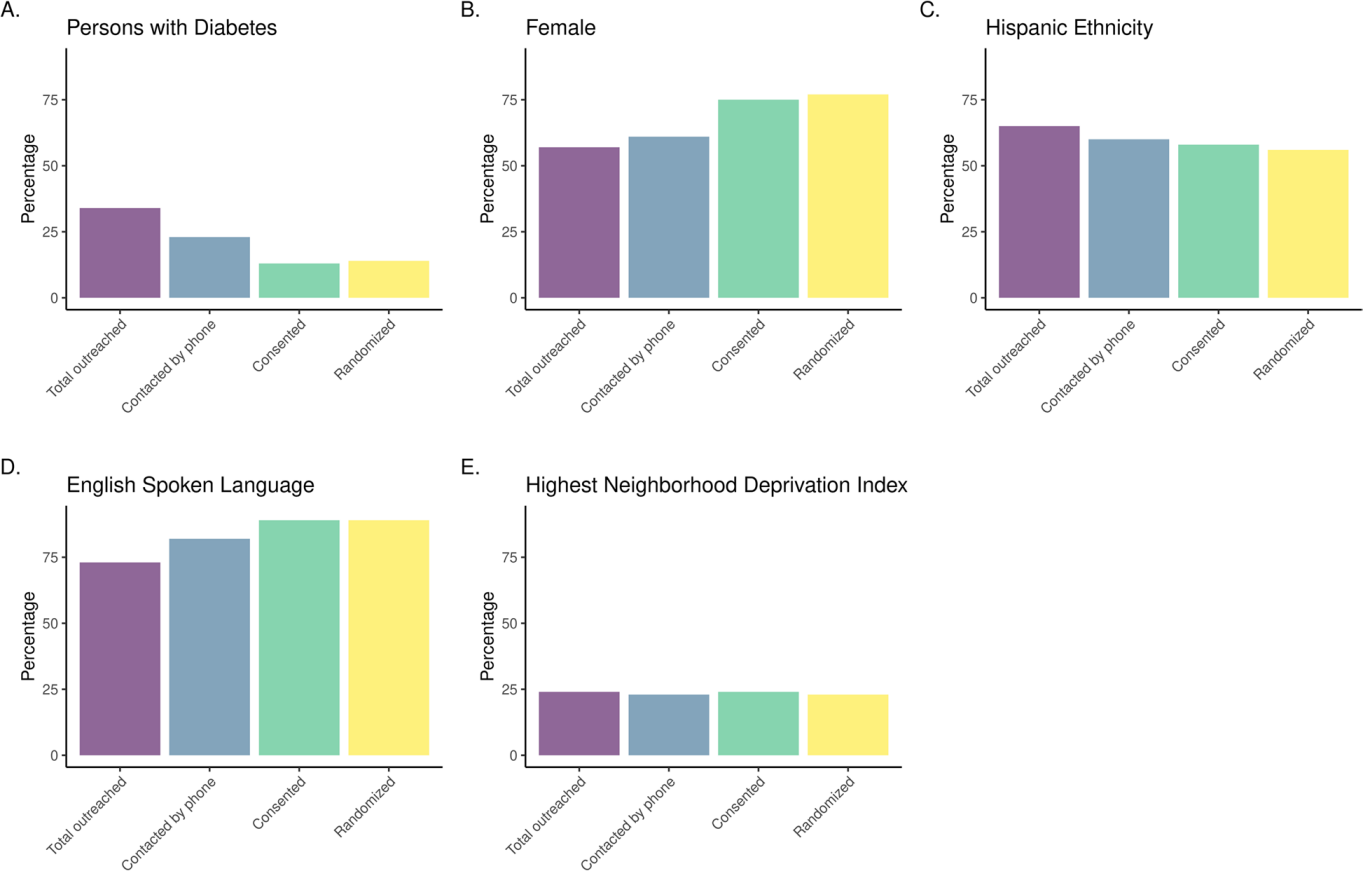
Display of differences across sociodemographics throughout the recruitment process

Figure 1 displays the recruitment flow from those who were potentially eligible and sent an information message to those who were ultimately randomized. Figure 2 summarizes the demographic characteristics of patients as they moved through the recruitment process. It illustrates how people with diabetes and of Hispanic ethnicity were less likely, and those who were female and preferred English as their spoken language were more likely to be ultimately randomized. Neighborhood factors, based on the NDI, were not different across recruitment subgroups.

## Discussion

Using electronic health records to identify potential participants for eligibility to be in a randomized physical activity behavior trial and subsequently attempting to recruit them by phone, we found demographic differences from the period of initial outreach to the point of randomization. At each stage of recruitment, patients with diabetes, males, patients of Hispanic ethnicity, and with a Spanish speaking preference were less likely to move to the next stage. While there were slight differences in the neighborhood deprivation index, our recruitment strategy appeared to successfully retain patients living in the most deprived neighborhoods. In sum, our strategy of initial email outreach followed by telephone outreach and screening appeared, overall, to result in a demographically diverse cohort, albeit with an overrepresentation of patients with prediabetes rather than diabetes and females over males.

There is a paucity of studies that have examined demographics as patients flow through a recruitment process. Mazzeo and colleagues examined enrollment of families in a pediatric obesity intervention and reported on demographic information through recruitment flow [11]. They did not find any sex, race, or ethnicity differences during the process of interested families calling for information, phone screen eligibility, and attendance at baseline to the point of study enrollment. In contrast, we found differences across recruitment stages. Mazzeo et al.’s recruitment strategy used passive methods in which interested people reached out to study staff and, after eligibility was confirmed, families were invited to in-person screenings. Our active recruitment strategy, with emails sent to potentially eligible adults, followed by telephone outreach, preserved the demographics of the underlying eligible population that allowed us to study which characteristics were associated with remaining interested in participating. While patients with certain demographic characteristics were less likely to continue, we ultimately enrolled a cohort with high sociodemographic diversity.

Of note, 76% of randomized participants were either Hispanic or Black, a percentage similar to those who were sent outreach email or mail (77%). Investigators from the Lifestyle Intervention for Treatment of Diabetes (LIFT Diabetes) trial used EHRs, as well as other strategies, to recruit participants into their trial of two contrasting lifestyle-focused interventions [12]. Using all recruitment methods, they found that their yield was higher for White compared with Black or Hispanic adults. When examining yield by the EHR as the recruitment method, they found 72.9% of Whites but only 49.6% of Blacks were randomized [12]. Their screening methods required in-person clinic visits, which were more intensive than our mail-based methods. Perhaps reducing the intensive requirements of screening may increase racial and ethnic representation into lifestyle behavior trials.

It was not surprising that females were more likely to be randomized than males. In a review of 244 publications of randomized lifestyle interventions of diet, exercise, or both, Pagoto and colleagues found that males were about 27% of the trials’ participants [13], similar to our findings. Reasons for this disparity are largely unknown, given that only about one quarter of the trials provided an explanation for the sex distribution, although a number of the trials evaluated were designed solely for females. Little information is provided on recruitment strategies for lifestyle trials, so guidance on which messages may appeal to males is not known [13].

Of all patients who were sent emails/mailings, 34% were people with diabetes; however, only 14% of patients randomized into the study had diabetes. Patients with diabetes were not only less likely to be randomized, they were also less likely to be reached by telephone outreach. These patients met eligibility criteria based on the code that was run against the EHR, but they may not have considered themselves in sufficiently good health to participate and did not respond to our telephone messages. For patients interested in participating, we required a primary care provider to confirm that there were no contraindications to increasing physical activity. During the time needed to obtain this approval, it is possible that some patients lost interest.

Nobles and colleagues conceived a conceptual framework of engagement in weight management programs that ranges from recruitment to program completion [3]. The enrollment stage is considered to include the recruitment to initiation phases or, in the case of trials, randomization. They differentiate intenders, or those who intend to participate, from initiators, or those who do participate in the program. For this study, the intenders can be defined as patients who consented to participate (*N* = 721), which is a much larger group compared with those who were randomized (*N* = 451). Nobles et al. suggest that intenders may have internal or external barriers that may require individualized tailoring [3]. Barriers that may have discouraged patients who consented but were not randomized include the requirements of completing baseline assessments, other commitments that arose, being available for motivational interviewing calls if randomized to that arm, and general loss of interest in participating. Additionally, because all study aspects were remotely delivered, patients and staff had no opportunity to interact face-to-face. The commitment may have been lower due to feeling minimal obligation to another person.

There are strengths and limitations to this study. Strengths include the ability to reach out to a large number of potentially eligible patients, the sociodemographic diversity of the potentially eligible patients, and systems in place to be able to capture sociodemographic data throughout the recruitment process. Recruiters, measurement staff, and intervention staff were bilingual and bicultural, which was another strength. Limitations include that study results are only applicable to patients with prediabetes or diabetes not prescribed insulin, patients of the KPSC healthcare system, and those who had email addresses and telephone numbers available in the EHR. We did reach out via mail for patients without email addresses, but low response rates and its additional costs suggested this method was not sustainable. We did not have in-person contact with patients, which may have contributed to less interest in trial participation. There are other potential barriers to participation in the trial, such as the time commitment required to complete data collection and attend motivational interviewing sessions (if randomized to that arm), managing personal and work obligations, and dealing with concomitant health conditions, that we were not able to assess. The need to obtain rigorous data on physical activity using accelerometers may have lowered the response rate but would not be needed if the motivational interviewing intervention was implemented in real practice. We cannot speculate whether our results would be replicable in trials outside the realm of behavior change interventions, such as pharmacological or device clinical trials.

## Conclusions

The recruitment strategies that we employed resulted in people with diabetes, males, Hispanics, and Spanish speakers were less likely to be randomized. However, the trial was successful in enrolling a majority of participants reporting to be of Hispanic ethnicity. Neighborhood deprivation did not differ across stages. Nonetheless, identifying potential patients from EHR resulted in sociodemographically diverse participants.

## Supplementary Information

Supplementary Material 1.

## Data Availability

The datasets generated and analyzed during the study are available from the corresponding author on reasonable request.
